# Analysis of the gel properties, microstructural characteristics, and intermolecular forces of soybean protein isolate gel induced by transglutaminase

**DOI:** 10.1002/fsn3.2706

**Published:** 2022-01-14

**Authors:** Zhanrui Huang, Jing Sun, Liangzhong Zhao, Wanying He, Teyuan Liu, Binbin Liu

**Affiliations:** ^1^ College of Food and Chemical Engineering Shaoyang University Hunan Provincial Key Laboratory of Soybean Products Processing and Safety Control Shaoyang China; ^2^ Jinzai Food Group Co., Ltd. Yueyang China; ^3^ Pingjiang Jinzai Food Co., Ltd Yueyang China

**Keywords:** gel properties, intermolecular forces, microstructural characteristics, soybean protein isolate, transglutaminase

## Abstract

Soybean protein isolate (SPI) is a high‐quality plant protein that is primarily used to process various soybean products coagulated by transglutaminase (TGase). In this study, the degree of hydrolysis (DH), sulfhydryl content (SH), surface hydrophobicity (*H_0_*), secondary structural constitution, and microstructure of TGase‐treated soybean protein (SPI, 7S, and 11S) were determined, as well as the effects of NaCl, urea, and SDS on the properties and intermolecular forces of SPI gel were analyzed. The results show that the *H_0_* and SH content of SPI, 7S, and 11S decreased significantly with TGase treatment time (*p* < .05), while the DH gradually increased and reached its highest value (3.72%, 7.41%, and 1.27%, respectively) at 30 min. As the concentration of these two secondary structures exhibited an inverse relationship, the degradation of β‐turns resulted in the increase in β‐sheets. The microstructures of SPI and 11S gels were similar, being denser and more ordered than 7S gel. The low concentration of NaCl solution (0.2 mol/L) enhanced gel properties and intermolecular forces, promoting the formation of SPI gel, whereas a high concentration (0.4–0.8 mol/L) had a significant inhibitory effect. Urea and SDS solutions substantially inhibited the formation of SPI gel, leading to significant decreases in the water holding capacity and hardness as well as a considerable increase in the coagulation time (*p* < .05). The results revealed that hydrogen bonds and hydrophobic interactions were the main intermolecular forces responsible for the gel formation. This study provides adequate technical support and a theoretical basis for soybean protein gel products.

## Graphical abstract

The gel properties of SPI, 7S, and 11S were similar when treated by treated with TGase, whereas the microstructures of SPI and 11S gels were denser and more orderly than 7S gel. Salt, urea, and SDS all had a significant effect on the formation of SPI gel. Hydrogen bonds and hydrophobic interactions were the main forces in SPI gel.

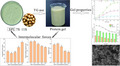

## 1 INTRODUCTION

Soybean protein isolate (SPI) is a high‐quality plant protein produced from defatted low‐temperature soybean meal. It consists of over 90% protein and eight essential amino acids (with digestibility and utilization rates of 93%–97%), which is close to or higher than the ideal composition recommended by the Food and Agriculture Organization (FAO) and the World Health Organization (WHO) (Preece et al., 2017; Zheng et al., 2019). According to the different sedimentation coefficients of protein at pH 7.6 and ionic strength of 0.5 mol/L, the main components of SPI can be divided into 2S, 7S, 11S, and 15S, of which 7S (β‐conglycinin) and 11S (glycinin) account for 80% of the total content (He et al., 2021; Zhao et al., 2007).

The remarkably high nutritional value and numerous functional characteristics of SPI have widely promoted its application in the food industry, where it can increase flavor, prevent juice separation, improve the quality of meat products, and prolong the shelf‐life (Fang et al., 2021; Wu et al., 2017). Researchers have found that adding SPI can improve the color, stability, and rheology of minced pork, reduce animal fat intake, and improve the product's taste (Li, Chen, et al., 2020; Li, Sukmanov, et al., 2020; Paglarini et al., 2018). Gelation is one of the most important aspects of the functional properties of SPI (Fang et al., 2021). When heated, SPI can form a gel, solidifying a protein matrix and forming a network structure with a certain intensity (Li, Chen, et al., 2020; Li, Sukmanov, et al., 2020; Wan et al., 2021). Many studies have shown that the thermal aggregates of SPI are intermediate products of gel formation, and their shape, size, and solubility have considerable influence on the structural and functional properties of SPI gel. Moreover, the morphology and quantity of thermal aggregates are affected by many factors, such as temperature, ionic strength, pH, protein composition, and protein concentration (Niu et al., 2017; Wang et al., 2018; Xu et al., 2021).

Protein gel is formed by balancing protein–protein and protein–water interactions (Li et al., 2019). Many scholars have found that the acting forces responsible for the formation and maintenance of the gel structure include electrostatic forces, hydrogen bonds, hydrophobic interactions, ionic bonds, and disulfide bonds (Li et al., 2019; Nivala et al., 2021; Renkema et al., 2001). Electrostatic forces and hydrophobic interactions are the main forces that promote protein aggregation and gel formation. Moreover, electrostatic repulsion between protein molecules affects the aggregate morphology (Wang et al., 2014). In addition, studies have shown that NaCl can neutralize and break ionic bonds, urea can destroy the formation of hydrogen bonds, and sodium dodecyl sulfate (SDS) can inhibit the hydrophobic interactions between proteins molecules (Eissa, 2019; Wang et al., 2018). Therefore, soybean producers are keen to investigate the impact of various environmental conditions on the gelation of SPI.

Transglutaminase (TGase) is a transfer enzyme that catalyzes the acyl transfer reaction and can also catalyze the dissociation and denaturation of proteins. This exposes diverse functional groups between protein molecules and promotes the chemical bonding of adjacent protein molecules, resulting in the formation of a tertiary network structure gel (Nivala et al., 2021; Xu et al., 2021). TGase‐modified soybean protein can improve its gel properties, hydrophobicity, solubility, and texture, resulting in a high yield, high quality, and appealing taste (Gao et al., 2021; Qin et al., 2016; Xu et al., 2021). In addition, researchers have reported the effects of TGase from different sources (e.g., *Streptomyces mobaraensis* and *Bacillus subtilis*) on the properties of SPI gel at different PH and temperatures (Liu et al., 2020). They have also tried to modify the protein by TGase collocation with microfluidization (Hu et al., 2021). However, the previous studies have focused on the properties of soybean protein gelation catalyzed by plant protease, whereas the molecular force and coagulation mechanism of soybean protein solidified by TGase have not been reported.

In this study, the gel solutions of SPI, 7S, 11S were extracted and prepared using TGase‐coagulated soybean protein. Conventional physicochemical methods were used to determine the DH, SH, *H_0_*, secondary structural constitution, and microstructure of the soybean protein gel. Additionally, the effects of NaCl, urea, and SDS solutions on the properties and intermolecular forces of SPI gel were analyzed. The purpose of this study was to identify changes in gel properties, microstructural characteristics, and intermolecular forces of TGase‐coagulated soybean protein gel and provide adequate theoretical and technical support for developing high‐quality soybean protein gel products.

## 2 MATERIALS AND METHODS

### 2.1 Materials and chemicals

The defatted soybean meal was purchased from the Wanyue Import and Export Trade Co. Ltd. TGase was obtained from the Hunan Provincial Key Laboratory of Soybean Products Processing and Safety. All other chemical reagents were purchased from the Nanjing Chemical Reagent Co. Ltd.

### 2.2 Preparation of SPI, 7S globulin, and 11S globulin

**FIGURE 1:**
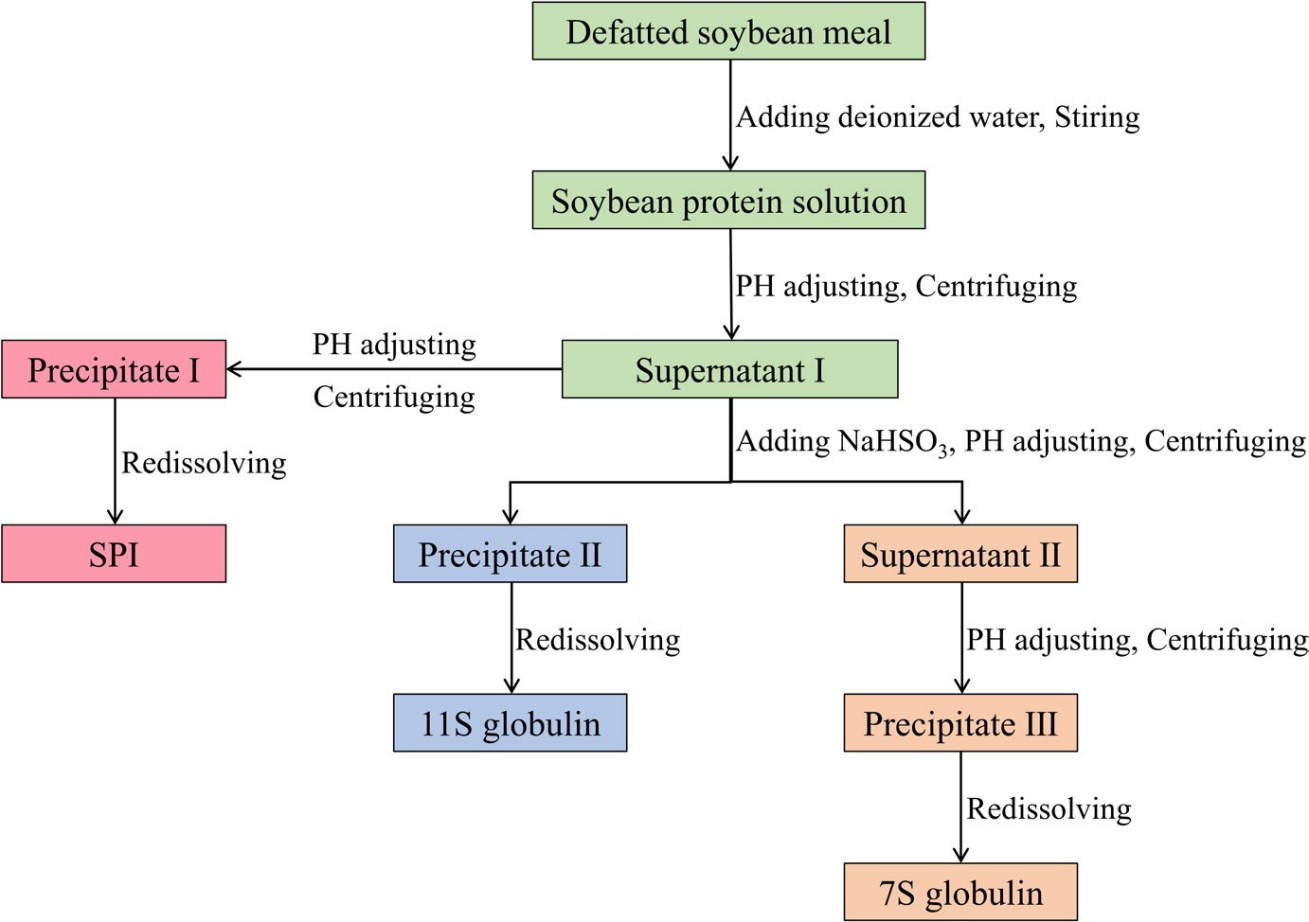
The flow extraction design of SPI, 7S, and 11S

SPI, 7S, and 11S were prepared using the methods described by Nagano and Tokita (2011) (Figure 1). Defatted soybean meal (200 g) was dissolved in 2 L deionized water, and the pH was adjusted to 8.0 with 2 mol/L NaOH. The mixture was stirred at a low speed for 2 h at room temperature (MYP11‐2A, Shanghai Meiyingpu Instrument Manufacturing Co., Ltd) and centrifuged at 3578 *g* for 20 min (VELOCITY18R, Dynamic). The pH of supernatant I was adjusted to 4.5 with 2 mol/L HCl to precipitate the SPI. The collected precipitate I protein was redissolved in deionized water, yielding an SPI solution with a protein content of 7%.

The supernatant I was added to NaHSO_3_ solution (until the concentration of SO_3_^2−^ was 0.01 mol/L), the pH adjusted to 6.4 with 2 mol/L HCl, stored at 4℃ for 8 h, and the mixture was centrifuged at 7156 *g* for 20 min. The collected precipitate II protein was redissolved in deionized water, resulting in an 11S globulin solution with a protein content of 7%.

Supernatant II was added to NaCl solution (until the concentration of Cl^−^ was 0.25 mol/L), the pH adjusted to 4.8 with 2 mol/L HCl, and centrifuged at 7156 *g* for 30 min. The collected precipitate III protein was redissolved in deionized water to produce 7S globulin solution with a protein content of 7%.

SPI (100 ml), 7S globulin, and 11S globulin solutions were heated at 95℃ for 10 min, then cooled to 55℃ before adding TGase (0.5%, v/m). After that, the gel properties were determined at 0, 5, 10, 15, 20, 25, and 30 min, respectively.

### 2.3 Degree of hydrolysis

The degree of hydrolysis (DH) in protein solutions was calculated based on the determination of free amino groups by the o‐phthaldialdehyde (OPA) method (Perna et al., 2020) with the following modifications: 200 µl protein gel solution was added to 3.8 ml OPA reagent and incubated at 25℃ for 2 min before measuring absorbance at 340 nm with an ultraviolet spectrophotometer (UV‐1780). Serine was used for the standard curve, while unhydrolyzed sample served as the blank. Each group was measured in triplicate.

### 2.4 Surface hydrophobicity (*H_0_*)

According to Zhang et al. (2020), the *H_0_* of protein solutions was determined. The protein solutions were diluted with phosphate buffer solution (0.1 M, pH 6.8) to form different concentration (0.02%, 0.04%, 0.06%, and 0.08%). Eight milliliter of each protein diluent was transferred to new test tubes and 20 µl ANS (8‐Anilino‐1‐naphthalenesulfonic acid) was added as a fluorescent probe and mixed thoroughly. After 30 min of light protection of the samples, the fluorescence intensity was measured with a fluorescence spectrophotometer (F‐4500) at excitation and emission wavelengths of 390 nm and 470 nm, respectively, with a slit of 5 nm. The *H_0_* was the slope obtained by taking the measured fluorescence intensity as the ordinate and the protein concentration as the abscissa. Each group was measured in triplicate.

### 2.5 Sulfhydryl content

The free sulfhydryl content (FSH) of protein solutions was determined according to Wan et al. (2021). The protein concentration of the protein solutions was adjusted to 2 mg/ml with Tris‐Gly buffer solution (0.086 mol/L Tris, 0.09 mol/L Glycine, 4 mmol/L Na_2_EDTA, pH 6.8), and then centrifuged at 8000 r/min for 15 min. The supernatant was immediately mixed with Ellman's reagent (4 mg of DTNB/mL of phosphate buffer solution) at a 100:1 ratio and stored in the dark for 15 min. The absorbance values were measured using an ultraviolet spectrophotometer (UV‐1780, Shimadzu, Japan) at 412 nm. Eight mol/L urea and 0.5% SDS were added to the Tris‐Gly buffer solution, and the total sulfhydryl content (TSH) was determined by the same method. The Tris‐Gly buffer solution without protein was used as the blank. Cysteine was used for the standard curve, and each group was measured in triplicate.

### 2.6 Secondary structural constitution

The secondary structural constitution of protein solutions was determined using circular dichroism (CD) spectra (J‐815, Jasco Corp), according to Zheng et al. (2019). The protein concentration of the solutions was adjusted to 0.1 mg/ml with phosphate buffer solution (0.1 M, pH 6.8) and centrifuged at 7156 *g* for 20 min. The CD spectrum of the sample was detected in a 1 mm quartz CD cuvette at 20℃, with a scanning wavelength range of 190–250 nm, a resolution of 1 nm, a bandwidth of 1 nm, a scanning speed of 50 nm/min, a reflection time of 1 s, and four scanning times. Each group was measured in triplicate.

### 2.7 Scanning electron microscopy

The microstructures of SPI, 7S globulin, and 11S globulin gels were examined using scanning electron microscopy (*SEM*; JSM‐8360LV, JEOL), according to a modified version of the method described by Wang et al. (2018). The samples were cut into small pieces (2 × 2 × 1 mm) and they were subjected to a pretreatment process that included fixing, dehydration, defatting, and freeze‐drying. The dried samples were operated at 5.0 kV for observation.

### 2.8 Gel characteristics and intermolecular forces

Ten milliliters of SPI solutions were heated to 95℃ for 10 min, cooled to 55℃, and TGase (0.5%, v/m) was added. Then, various concentrations of NaCl solution (0, 0.2, 0.4, 0.6, 0.8 M), urea solution (0, 0.2, 0.4, 0.6, 0.8 M), and SDS solutions (0, 0.2, 0.4, 0.6, 0.8 M) were added. The DH, coagulation time, WHC, and texture characteristics of SPI gels were measured. The DH of SPI was determined according to section 2.3. The time at which SPI gel had a fixed shape and no fluidity was the coagulation time. The WHC of SPI was determined by drying to constant weight method (Wang et al., 2018).

The texture (hardness) of SPI gels was measured in triplicate using a texture analyzer (LS‐5, AMETEK) at four corners and the center of the gel (Sun et al., 2019). The texture analyzer probe model was P/0.5, whereas TPA was the test mode. The speeds for pretest, test, and posttest were 2.0, 1.0, and 1.0 mm/s, respectively. The compression distance was 4 mm, the interval time was 2.0 s, the trigger type was Auto, and the data acquisition rate was 50.0 PPS.

### 2.9 Statistical analysis

The data were presented by the mean ±standard deviation (*SD*). SPSS 22.0 and origin 9.1 were used for statistical analysis and analysis of variance (ANOVA). To identify significant differences among groups, significant and extremely significant levels were chosen at *p* <.05 and *p* <.01, respectively.

## 3 RESULTS AND DISCUSSION

### 3.1 Changes in the DH and surface hydrophobicity of TGase‐treated soybean protein

**FIGURE 2:**
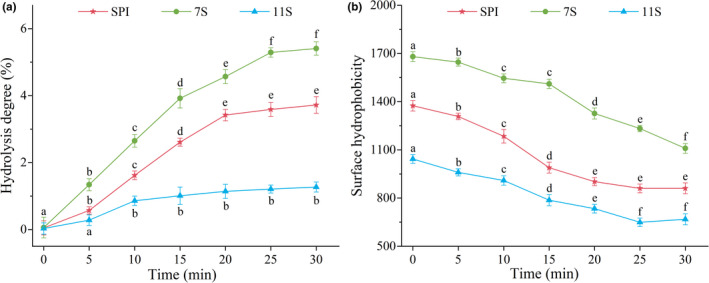
Changes in the degree of hydrolysis and surface hydrophobicity of SPI, 7S, and 11S treated by Tgase. The lowercase letters above the columns represent statistically significant differences between groups (*p* <.05)

The DH of SPI, 7S, and 11S gradually increased with treatment time, reaching 3.72%, 5.41%, and 1.27%, respectively, in 30 min (Figure 2a). This may indicate that the 11S gel solution was more stable than SPI and 7S solutions. On the one hand, it is possible that the 11S subunits produced dimers during the gel process, and the dimers combined to form stronger and more stable quaternary structures. However, the amino acid residues were buried in the hydrophobic region, and the DH of amino acid was relatively small (Yang, Wang, et al., 2021). Renkema et al. (2001) reported that the strength of 11S globulin thermal gel was greater than the 7S, indicating that the 11S globulin molecule contained more disulfide bonds and fewer thiol/disulfide bond exchanges. In addition, Wu et al. (2015) revealed that the protein concentration released by heat‐induced soybean protein gel increased steadily in the initial 0.5–48 hr, but there was no significant change from 48 to 56 hr. This was similar to the results of this study, which verified that the DH of protein was positively correlated with solubility.

The *H_0_* of SPI, 7S, and 11S treated by TGase decreased significantly (*p* <.05), from 1374.62, 1680.07, and 1043.68 to 860.68, 1109.24, and 667.95, respectively (Figure 2b). In particular, the changes in *H_0_* for SPI and 11S were similar, initially decreasing remarkably from 0 to 20 min (*p* <.05) and then remaining steady from 25 to 30 min (*p* >.05). This may be because TGase catalyzed the hydrophobic binding of the protein hydrophobic terminal residues and the hydrophobic side chains exposed by proteolysis as well as the gel structure formed by the aggregation of hydrophobic binding protein molecules that encircled the hydrophobic groups and reduced their hydrophobicity (Nivala et al., 2021; Xu et al., 2021). Moreover, 7S had a greater *H_0_* than SPI and 11S, which may be explained as follows: (1) the molecular weight of 7S globulin was lower than that of 11S globulin, whereas the hydrophobic surface region of 7S globulin was more than that of 11S globulin. This may have resulted in 7S having a higher *H_0_* (Renkema et al., 2001; Wang et al., 2014), and (2) the aggregation of acidic and basic peptides of 11S globulin inhibited the improvement of *H_0_* (Sorgentini et al., 1995).

### 3.2 Changes in the sulfhydryl content of soybean protein treated by TGase

**FIGURE 3:**
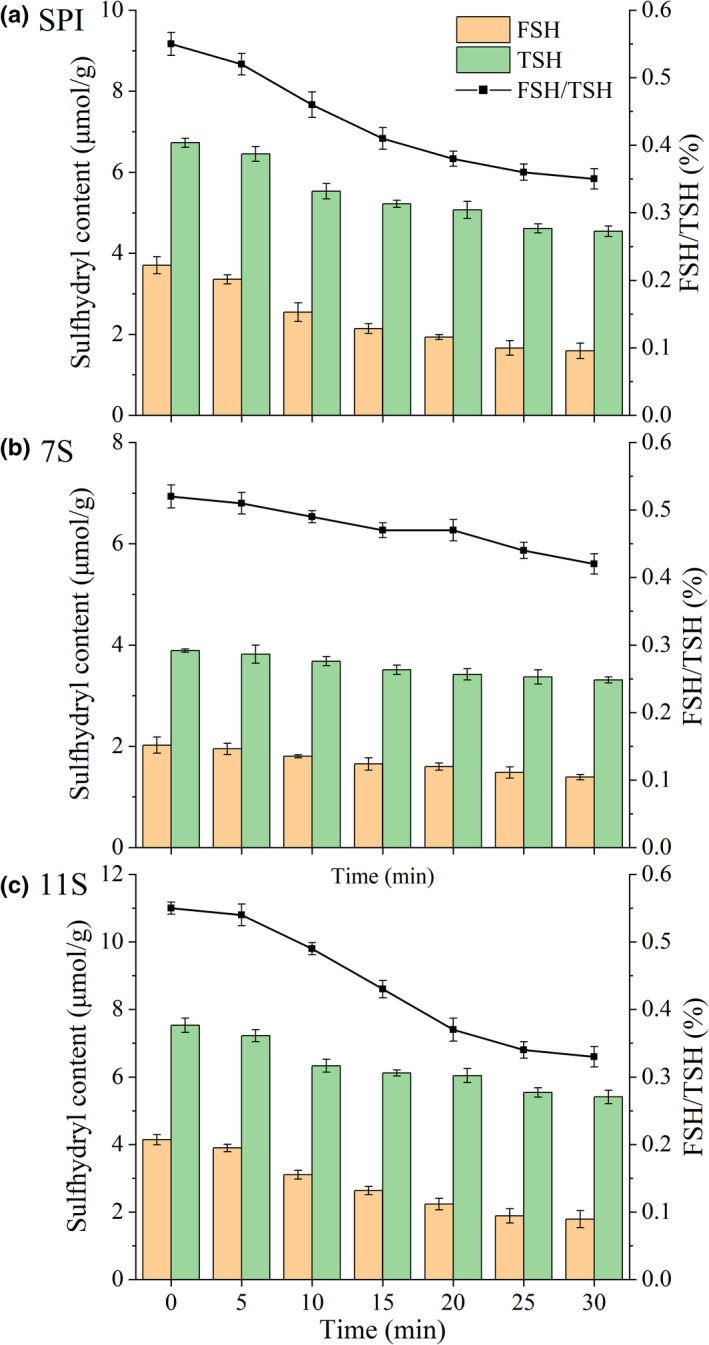
Changes in the sulfhydryl content of SPI, 7S, and 11S treated by Tgase

After different treatment times, the change in FSH content for SPI protein was the same as that of TSH, decreasing from 3.70 μmol/g and 6.73 μmol/g to 1.59 μmol/g and 4.54 μmol/g, respectively (Figure 3a). Previous studies have found that the sulfhydryl group of soybean protein exposed readily at high temperatures, oxidized to form disulfide bonds, and formed aggregates by hydrophobic interaction, thereby enhancing the strength of the protein gel (Huang et al., 2021). This was consistent with the conclusions of Puppo et al. (2004), which show that heating, extreme pH, and enzyme catalysis induced denaturation of soybean protein, resulting in a decrease in FSH and TSH content.

With an increase in treatment time, the changes in FSH and TSH content for 11S and SPISWZ decreased gradually (Figure 3c). However, the content of FSH and TSH in 7S had a little overall effect (Figure 3b), possibly due to the low amount of SH in 7S being less affected by TGase and coagulation time (Yang, Deng, et al., 2018). The ratio of FSH to TSH reflects the degree of unfolding in the protein tertiary structure. The higher the ratio, the stronger the degree of unfolding (Yang, Lei, et al., 2018). In this study, the ratios of FSH to TSH for SPI, 7S, and 11S treated by TGase were significantly reduced, indicating that the degree of unfolding in the tertiary structure of soybean protein was weak. This may be because TGase inhibited the expansion of protein molecules when inducing gel formation (Gao et al., 2021).

### 3.3 Changes in the secondary structural constitution of soybean protein treated by TGase

**FIGURE 4:**
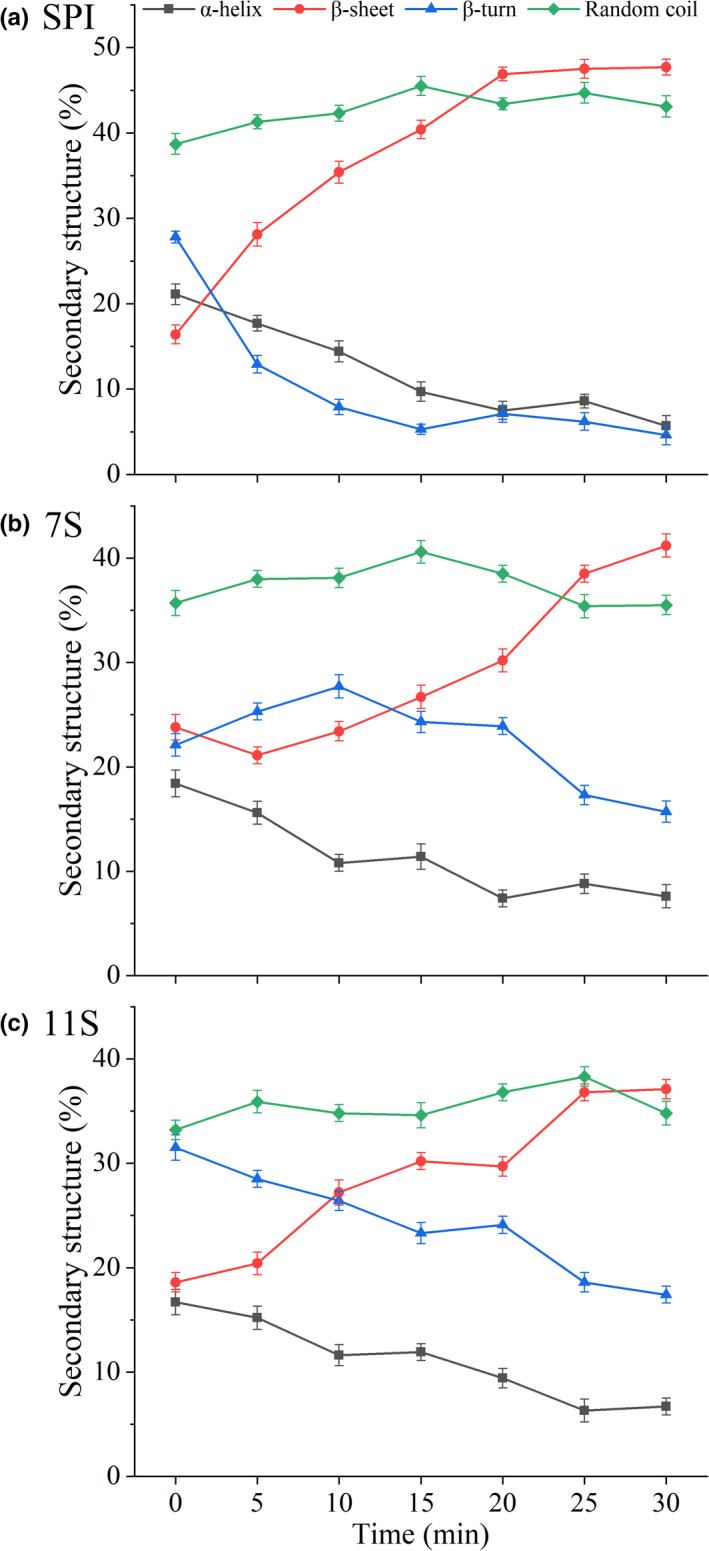
Changes in the secondary structural constitution of SPI, 7S, and 11S treated by Tgase

The changes in the secondary structure constitution in SPI, 7S, and 11S treated by TGase were generally consistent (Figure 4). α‐Helices decreased significantly with an increase in treatment time, from 21.12%, 18.40%, and 16.71% to 5.71%, 7.63%, and 6.69% for SPI, 7S, and 11S, respectively. This may be because TGase can catalyze the conversion of SH into disulfide bond and the formation of protein intermolecular aggregates, which was verified by the significant decrease in SH content. This conclusion has also been confirmed by Qin et al. (2016) and Wang et al. (2013). Studies have shown that different denaturation methods were closely related to changes in α‐helix and random coil content in soybean protein (Wu et al., 2010; Xue et al., 2013). In particular, unfavorable environments can destroy the protein gel structure and secondary structural constitution, forming a large number of random coils (Zheng et al., 2019).

The content of β‐turns in SPI, 7S, and 11S decreased significantly with an increase in treatment time, from 16.40%, 21.12%, and 18.61% to 47.68%, 41.22%, and 37.10%, respectively. On the contrary, the β‐sheet content increased significantly with an increase in treatment time. This may be due to the degradation of β‐turns leading to the increase in β‐sheets as the concentration of these two secondary structures has an inverse relationship, as reported by Du et al. (2020). It has been reported that the increase in the β‐sheet structure that occurs after long‐term heating was related to the aggregation of α′ and α‐7S subunits (Petruccelli & Anon, 1995). Lee et al. (2002) reported that the increase in β‐sheets in the secondary structure of protein aggregates may be attributed to the relatively large and ordered hydrogen bonding surface area. Therefore, the secondary structure of SPI, 7S, and 11S induced by TGase may provide clues to their partial unfolding and aggregation that is attributed to the binding of soybean protein gels by TGase through two disulfide and hydrophobic bonds. This explanation was supported by the results of the surface hydrophobicity and sulfhydryl content of soybean protein treated by TGase.

### 3.4 Changes in the microstructure of soybean protein treated by TGase

**FIGURE 5:**
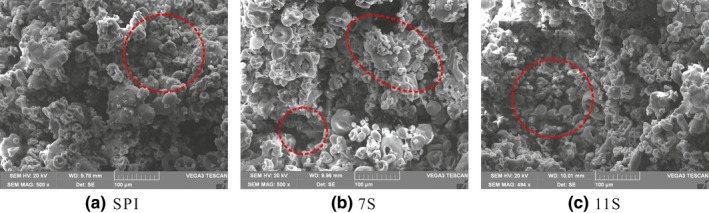
Changes in the microstructure of SPI, 7S, and 11S gel treated by Tgase

To further confirm the changes in the gel network of soybean protein catalyzed by TGase, the microstructure of SPI, 7S, and 11S gel was studied by scanning electron microscopy (*SEM*) (Figure 5). The *SEM* results show that the gel structure of 7S gel was loose, the random aggregation of protein molecules was discontinuous, and the pores were large and irregular. The gel structure of SPI was smooth, and the pore size decreased. The gel structure of 11S was the most compact, and the protein molecules were regularly aggregated and ordered. The microstructure results strongly support the speculation that the polymers formed by different soybean protein components during "preaggregation" have different structures.

The spatial structures of 11S globulin and 7S globulin were different, resulting in different gel properties. The results of the *SEM* were consistent with the results of the protein DH and *H_0_*. Therefore, these results reveal a close relationship between the microstructure, the DH, and *H_0_* of soybean protein. The SH content and *H_0_* of SPI and 11S were lower than 7S, indicating that the protein molecules had stronger disulfide bonds and hydrophobic interactions, thus making the gel structure more orderly and denser. Moreover, this uniform and continuous gel network structure can prevent the amino acid hydrolysis, which further supports the lower results of the DH for SPI and 11S. The results of Wu et al. (2015) have confirmed this, reporting that the gel structure of soybean protein was closely related to the protein solubility. Niu et al. (2017) and Gao et al. (2021) also confirmed the lower SH content was related to *H_0_* and denser microstructure. Moreover, Luo et al. (2019) and Wu et al. (2017) found that TGase could limit the hydrophobicity of the SPI gel network, resulting in higher thermal stability and deformation resistance, which is also consistent with the results of this study. These results confirm that TGase can catalyze the formation of denser soybean protein gel structures, which can accommodate more protein and other soluble substances.

### 3.5 Effects of NaCl solution on gel properties and intermolecular forces of SPI treated by TGase

**FIGURE 6:**
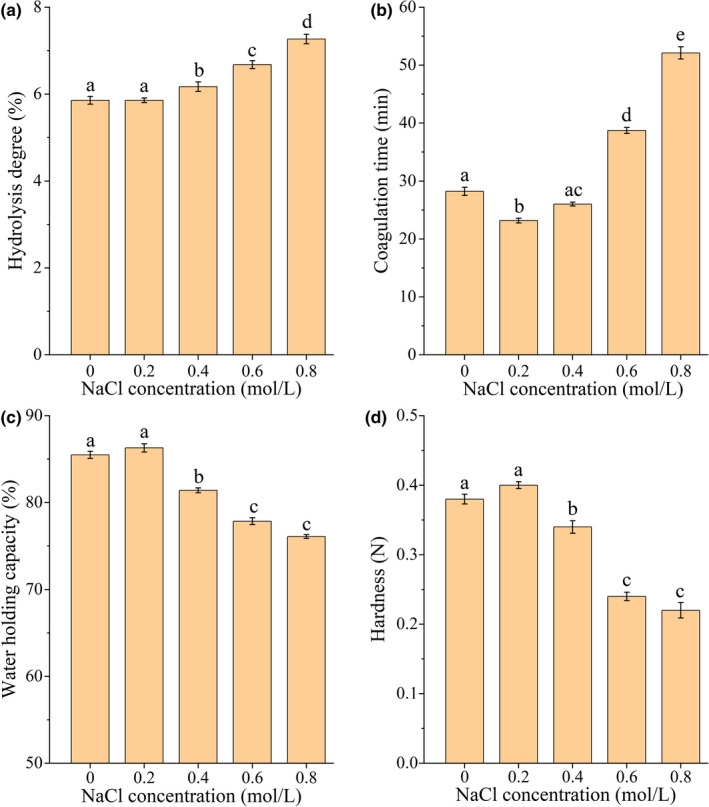
Effects of different concentrations of NaCl on the degree of hydrolysis, coagulation time, WHC, and hardness of SPI gel treated by TGase. A‐D represent the degree of hydrolysis, coagulation time, WHC, and hardness, respectively. The lowercase letters above the columns represent statistically significant differences between groups (*p* <.05)

With the increase in NaCl concentration, the DH of SPI increased from 5.86% to 7.27% (Figure 6a), indicating that NaCl could induce the densification and stability of SPI gel. The coagulation time of SPI gel decreased significantly at low concentrations of NaCl (0.2–0.4 mol/L) (*p* <.05). With the increase in NaCl centration, the gelation time of SPI protein gel increased gradually, and the coagulation time was 52.12 min at 0.8 mol/L (Figure 6b). The reason may be that lower concentrations of NaCl can neutralize the negative charge on the surface of SPI, reduce the electrostatic repulsion between peptide bonds, facilitate the proximity and condensation of peptide bonds, and shorten the formation time of SPI gel. At the same time, higher concentrations of NaCl can enhance the hydration of protein, destroy the secondary bonds between protein molecules, and prevent the protein molecules from aggregating together to form gel, thus increasing the coagulation time of SPI gel (Bi et al., 2013; Durand et al., 2002; Nishinari et al., 2014).

Studies have shown that the textural properties of soybean protein gel are closely related to the WHC (Wan et al., 2021; Wang et al., 2018), and similar results have been observed in this study. There was no significant change in the WHC and hardness of SPI gel for the lower NaCl concentrations (0.2 mol/L) (*p* >.05) (Figure 6c‐d). With the increase in NaCl solution concentration, the WHC and hardness of SPI gel decreased significantly (*p* <.05) to 76.09% mol/L and 0.22 N at 0.8 mol/L NaCl solution, respectively. These findings may be due to the low Na^+^ environment enhancing the hydration capacity of proteins through the combination of hydrated Na^+^ and charged groups on protein molecules. When the NaCl concentration was high, Na^+^ may not only shield the electrostatic effect of protein and destroy the protein hydration layer but also induce the formation of hydrophobic interactions and salt bridge effects, which lead to a decrease in the WHC of protein gels (Moritaka et al., 1991; Wang et al., 2018).

### 3.6 Effects of urea solution on gel properties and intermolecular forces of SPI treated by TGase

**FIGURE 7:**
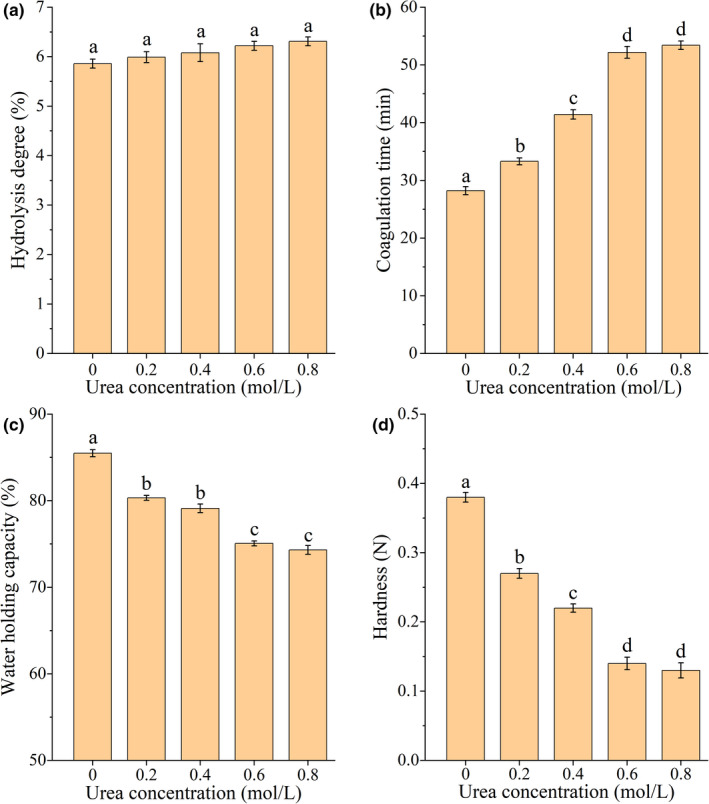
Effects of different concentrations of urea on the degree of hydrolysis, coagulation time, WHC, and hardness of SPI gel treated by TGase. A‐D represent the degree of hydrolysis, coagulation time, WHC, and hardness, respectively. The lowercase letters above the columns represent statistically significant differences between groups (*p* <.05)

As the concentration of urea increased, the DH of SPI gel increased slightly but not significantly (*p* >.05) (Figure 7a), indicating that urea did not affect the stability of the SPI protein gel. Meanwhile, the gelation time of SPI gel increased significantly (*p* <.05). This did not significantly change at high urea concentrations (0.6–0.8 mol/L), and the coagulation time was around 53 min (Figure 7b). The reason for this may be that urea molecules can form intermolecular hydrogen bonds rapidly with water in the SPI solution, destroying the structure of water and changing the polarity of the environment around protein molecules. This can then affect the hydrophobic interactions between protein molecules, leading to their expansion, the exposure of nonpolar groups, and thus inhibiting the formation of protein gels. These assumptions have been demonstrated by many researchers (Su & Dias, 2016; Zou et al., 1998). In addition, Bennion and Daggett (2003) found that 8 mol/L urea could change the structure and kinetics of water in the protein solution, reduce the hydrophobic interaction, and promote the solvation of hydrophobic groups, which indicates that urea affected SPI gel through direct and indirect mechanisms.

The WHC and hardness of SPI gel decreased significantly as the concentration of urea increased (*p* <.05), from 85.49% and 0.38 N to 74.31% and 0.13 N, respectively (Figure 7c‐d). The changes in the WHC and hardness revealed that the protein molecular structure of SPI was in a state of expansion after the denaturation of urea solution and TGase, and the quaternary, tertiary, or secondary structures changed. These structural changes were unfavorable to gel properties, which could be confirmed by the correlation between stability and texture properties of soybean protein gel and the WHC (Wan et al., 2021; Zhang et al., 2016).

### 3.7 Effects of SDS solution on gel properties and intermolecular forces of SPI treated by Tgase

**FIGURE 8:**
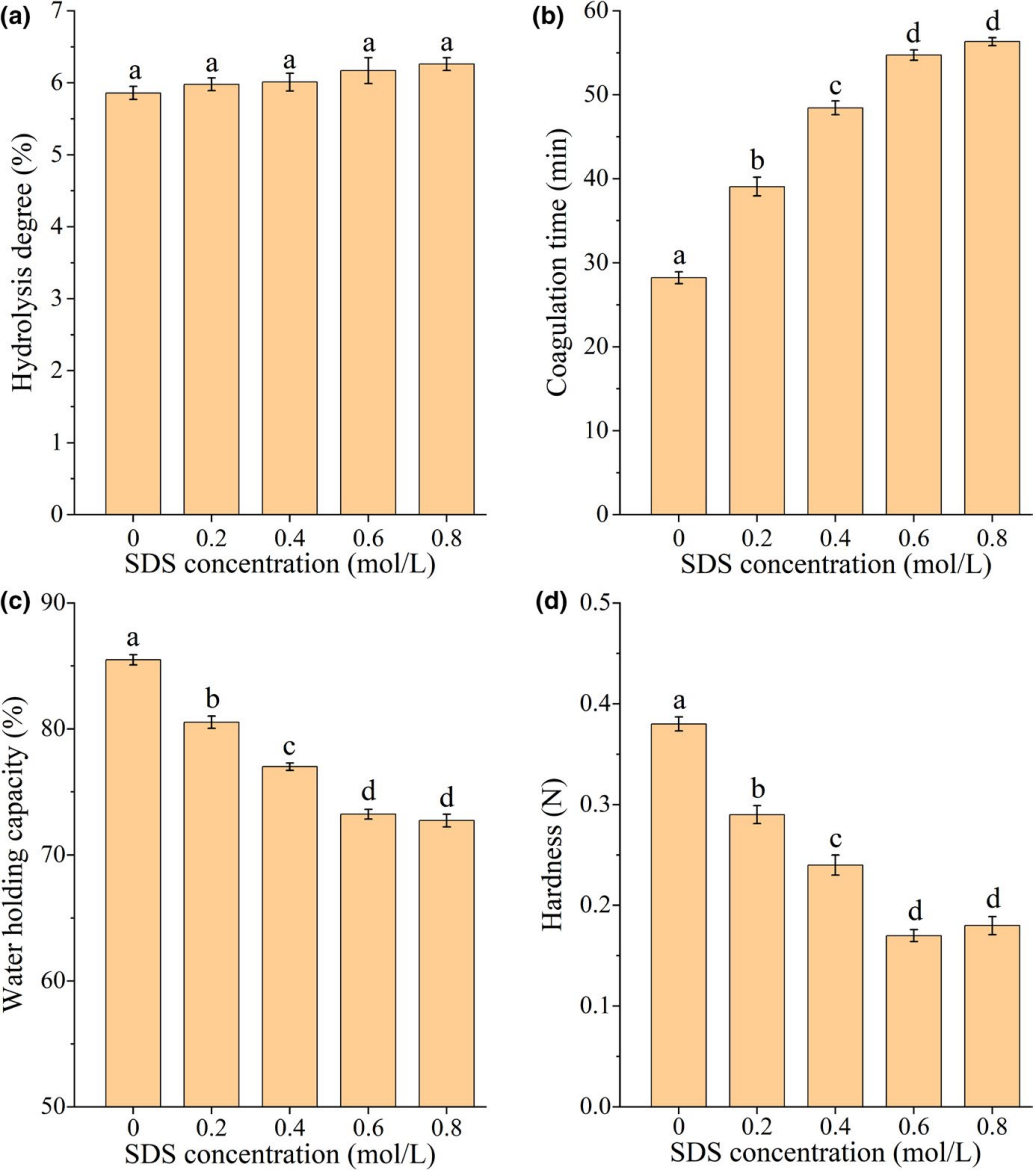
Effects of different concentrations of SDS on the degree of hydrolysis, coagulation time, WHC, and hardness of SPI gel treated by TGase. A‐D represent hydrolysis degree, coagulation time, WHC, and hardness, respectively. The lowercase letters above the columns represent statistically significant differences between groups (*p* <.05)

As the concentration of SDS increased, the DH of SPI gel increased slightly but insignificantly (*p* >.05) (Figure 8a), indicating that SDS did not affect the stability of SPI gel. The coagulation time of SPI in low SDS concentrations (0.2–0.6 mol/L) increased significantly (*p* <.05). When the concentration of SDS solution was greater than 0.6 mol/L, there was no significant change in the setting time of SPI gel (*p* >.05) (Figure 8b). This is because SDS is a hydrophilic surfactant that can destroy the hydrophobic binding of protein molecules, delay the formation of the SPI gel structure, and decrease the interfacial tension of proteins (Otzen, 2015).

The changes in the WHC and hardness of SPI gel with different SDS concentrations were consistent with those of urea, showing significant decreases (*p* <.05) for low SDS concentrations (0.2–0.6 mol/L) and no significant change (*p* >.05) for high SDS concentrations (0.6–0.8 mol/L) (Figure 8c‐d). A study by Eissa (2019) revealed similar results, indicating that SDS can dissociate the hydrophobic interactions between proteins and reduce the size of protein aggregates, thereby reducing the strength and viscosity of protein gels. Meanwhile, Lee et al. (2011) have shown that the decrease in protein gel strength and viscosity may be due to the presence of the SDS protein complex. Given the above findings, a reasonable explanation may be that the hydrophobic groups of SDS molecules combined with hydrophobic amino acids in a protein polymer, while the hydrophilic groups reacted with the positively charged amino acids on the other protein aggregates to produce ions. This may have resulted in the formation of a noncovalent crosslinking effect, which reduced the WHC and intensity of SPI gel (Eissa, 2019; Reynolds & Tanford, 1970).

## 4 CONCLUSIONS

The current study investigated differences in the physicochemical properties of soybean protein (SPI, 7S, and 11S) gels, and the effects of salt, urea, and SDS solution on the intermolecular forces of SPI gel coagulated by TGase. The results revealed that the gel properties of SPI, 7S, and 11S show similar changes with the increase in TGase treatment time, resulting in significantly lower *H_0_* and SH content and a gradual increase in the DH. The degradation of β‐turns led to an increase in β‐sheets as the concentration of these two secondary structures has an inverse relationship. The microstructures of SPI and 11S gel were similar, being denser and more orderly than 7S gel. Low concentrations of NaCl solution promoted the formation of SPI gel but inhibited gel formation at high concentrations, indicating that the electrostatic repulsion between protein molecules could be overcome. The addition of urea and SDS significantly inhibited or weakened the formation of SPI gel induced by TGase, which suggests that hydrogen bonds and hydrophobic interactions were the main intermolecular forces in SPI gel formation. This study, thus, provides effective technical support and a theoretical basis for soybean protein gel products.

## CONFLICT OF INTEREST

The authors declare that they have no conflict of interest in this study.

## AUTHOR CONTRIBUTIONS

**Zhanrui Huang:** Conceptualization (supporting); Funding acquisition (equal); Project administration (equal); Writing – original draft (supporting); Writing – review & editing (supporting). **Jing Sun:** Data curation (equal); Methodology (supporting). **Liangzhong Zhao:** Conceptualization (equal); Funding acquisition (supporting); Project administration (supporting); Supervision (equal). **Wanying He:** Software (equal); Validation (equal); Visualization (equal); Writing – original draft (equal). **Teyuan Liu:** Investigation (equal); Resources (equal); Visualization (equal). **Binbin Liu:** Data curation (equal); Investigation (equal); Resources (equal).

## Data Availability

Research data are not shared.
